# A novel ammoniation treatment of barley as a strategy to optimize rumen pH, feed degradability and microbial protein synthesis in sheep

**DOI:** 10.1002/jsfa.11205

**Published:** 2021-03-24

**Authors:** Alejandro Belanche, Ignacio Martín‐García, Elisabeth Jiménez, Nicholas N Jonsson, David R Yañez‐Ruiz

**Affiliations:** ^1^ Estación Experimental del Zaidín (CSIC) Granada Spain; ^2^ Institute of Biodiversity, Animal Health and Comparative Medicine, University of Glasgow Glasgow UK

**Keywords:** ammoniation, barley, feed utilization, rumen fermentation, sheep, urea

## Abstract

**BACKGROUND:**

Meeting the energy and nitrogen (N) requirements of high‐performing ruminants at the same time as avoiding digestive disturbances (i.e. rumen acidosis) is a key priority in ruminant nutrition. The present study evaluated the effect of a cereal ammoniation treatment, in which barley grains are combined with urea and enzymes that catalyze the conversion of urea to ammonia to optimize rumen function. Twelve rumen cannulated sheep were randomly divided into two groups and fed a diet containing 60% of ammoniated barley (AMM) or untreated barley supplemented with urea (CTL) to investigate the impact on rumen fermentation and feed utilization.

**RESULTS:**

AMM had higher total N content and effective rumen degradable N than untreated barely. AMM sheep had a consistently higher rumen pH throughout the day (6.31 *versus* 6.03) and tended to have a lower post‐prandial ammonia peak and higher acetate molar proportion (+5.1%) than CTL sheep. The rumen environment in AMM sheep favored the colonization and utilization of agro‐industrial by‐products (i.e. orange pulp) by the rumen microbes leading to a higher feed degradability. AMM sheep also had higher total tract apparent N digestibility (+21.7%) and urinary excretion of purine derivatives (+34%), suggesting a higher N uptake and microbial protein synthesis than CTL sheep.

**CONCLUSION:**

The inclusion of AMM in the diet of ruminants represents a valid strategy for maintaining rumen pH within a physiological range and improving N utilization by the rumen microbes, which could have positive effects on the health and productivity of animals in intensive production systems. These findings warrant further studies under conventional farm conditions. © 2021 The Authors. *Journal of The Science of Food and Agriculture* published by John Wiley & Sons Ltd on behalf of Society of Chemical Industry.

## INTRODUCTION

Modern intensive feeding systems demand large amounts of protein and cereal grains to support high production performances.^1^ However, dietary inclusion of large proportions of ground grains, especially those rich in starch that rapidly degrades in the rumen, can predispose livestock to digestive disorders such as rumen acidosis.^2^ In this sense, the inherent functional complexity of the rumen microbiota could represent an opportunity to find alternative energy (e.g. agro‐industrial by‐products) and nitrogen (N) sources (e.g. non‐protein N). The inclusion of agro‐industrial by‐products in the diets of ruminants has been described as a strategy for improving farm economic and environmental sustainability.^3^, ^4^ However, their practical use is often impaired by their low protein content and highly indigestible fiber.^5^ Alternatively, because up to 80% of the starch in the grains is rapidly degraded in the rumen,^6^ the partial substitution of protein (e.g. soybean meal) with non‐protein N, such as feed‐grade urea, may result in better synchronization on the availability of energy and N for the rumen microbes,^7^ as well as lower feeding cost. However, rapid hydrolysis of supplemented urea to ammonia in the rumen can override its utilization by the microbes leading to ammonia toxicity and increased feed N wastage in urine.^8^

Cereal ammoniation may represent a nutritional strategy that has not been studied sufficiently and could prevent these problems. Early studies showed that moist maize ammoniation preserved the whole grain satisfactory and led to a faster rate of digestion than untreated maize.^9^ Rode *et al*.^10^ reported improved values of starch digestibility in steers fed ammonia‐treated barley, but not with urea‐treated, compared to moist barley. Similarly, Robinson and Kennelly^11^ suggested that ammoniation of high‐moisture barley could cause a modification of the feed degradation kinetics favoring milk production in cows. These early studies reported some information about feed ammoniation, although information is still scarce when applied to modern concentrate diets.

Recent studies have investigated a more sophisticated ammoniation strategy in which cereal grains are moisturized and mixed with urea and enzymes able to catalyze the conversion of urea to ammonia.^12^, ^13^ This process was initially used to preserve cereal crops harvested with high moisture content, although these recent observations in commercial farms suggest that it can also be applicable to artificially moisturized grains.^12^, ^13^ This strategy can increase dietary N content, promote greater rumen pH and modulate the rumen microbiota, having positive effects on feed digestibility and performance and wellbeing in beef^13^ and dairy cows.^12^ However, the mode of action and the impact of such treatment on the rumen function and feed digestion kinetics are not fully understood.

We hypothesized that this new ammoniation treatment of grains could slow down the ammonia release in the rumen, compared to urea, leading to a better synchrony in the energy and N availability for the rumen microbes. Thus, the present study aimed to investigate whether cereal ammoniation could represent a strategy to prevent rumen acidosis and to improve rumen fermentation, microbial protein synthesis, feed microbial colonization and feed degradation kinetics (including agro‐industrial by‐products) in sheep.

## MATERIALS AND METHOS

### Animals and diets

**Figure 1:**
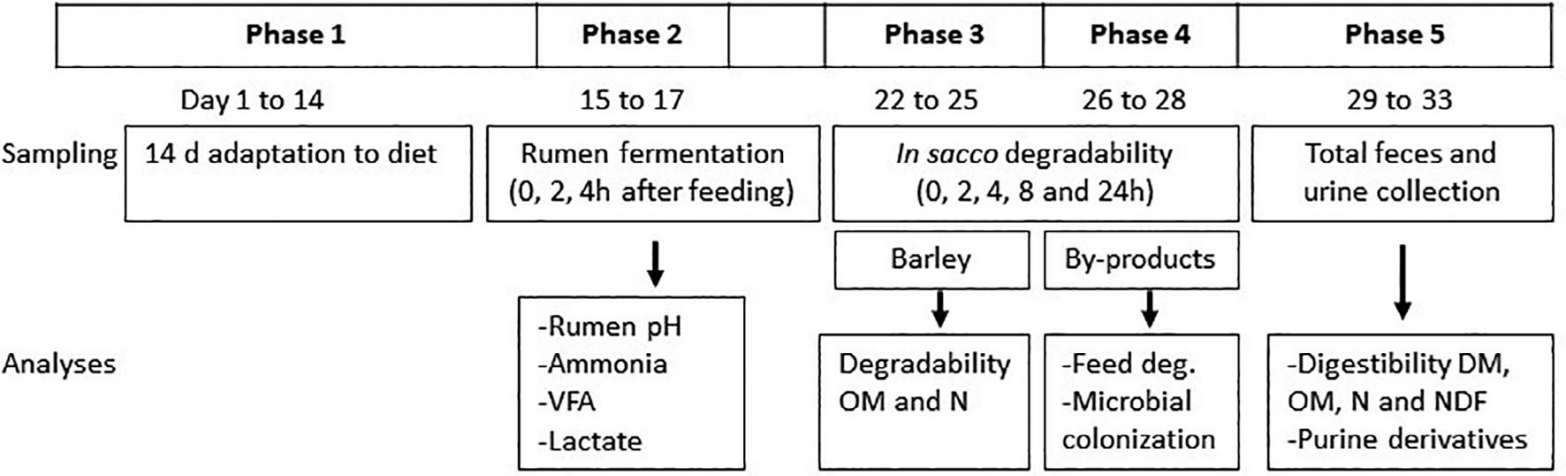
Diagram illustrating the experimental set‐up and analyses within each phase.

Animal procedures were conducted by trained personnel according to Spanish guidelines (RD 53/2013) and protocols were approved by the Ethical Committee for Animal Research (EEZ‐CSIC) regional government (3 September 2017). Twelve adult non‐pregnant Segureña sheep (53 ± 6.5 kg body weight) fitted with rumen cannulae were used in the study. Sheep were randomly allocated (*n* = 6) to one of the two experimental diets, which were kept constant throughout the entire duration of the study, according to a completely randomized design: sheep on the control diet (CTL) received a concentrate mostly based on untreated barley (and minority ingredients), whereas sheep on the ammoniated barley (AMM) diet received a similar concentrate in which the untreated barely was replaced by AMM. Both barleys had the same origin (Harbro Ltd, Turriff, UK) to discard potential bias in their initial chemical composition (Table 1). AMM was produced following an optimized commercial procedure consisting of mixing barley [980 g kg^−1^ dry matter (DM)], urea (15 g kg^−1^ DM) and Maxammon (5 g kg^−1^ DM), a product that contains enzymes to catalyze the conversion of urea to ammonia (Harbro Ltd). Water was added to the mixture to obtain a final moisture content of 200 g kg^−1^ and ingredients were thoroughly mixed for 5 min in a mixer wagon and finally stored under plastic sheeting at room temperature for a minimum of 14 days to facilitate the enzymatic conversion of urea to ammonia. Urea was added to the concentrate mix of the CTL diet to achieve similar total non‐protein N content in both diets. Both concentrate mixtures (CTL and AMM) were supplied by the same manufacturer and made from the same source and batch of cereal to avoid potential effects of variation in cereal composition. Diets offered to sheep consisted of oats hay and concentrate feed in a proportion 40:60, respectively, aiming to simulate a typical diet from intensive sheep production (chemical composition provided in Table 1). Sheep were fed once per day at 09.00 h and the feeding level was set to approximately 1.2 times the average maintenance energy requirements^14^ to prevent large differences in dry matter intake across animals. Forage and concentrate feeds were offered in separate feeders, which were weighed daily to assess the daily DM intake. The animal trial lasted 5 weeks and was divided into five phases, using the first two weeks (phase 1) for adapting sheep to the diets and the last three weeks (phases 2 to 5) for measurements as shown in Fig. 1.

**Table 1:** Ingredients and chemical composition in the concentrates and oats hay

Treatment^a^	CTL	AMM	Oats hay
Ingredients (g kg^−1^ DM)			
Untreated barley^b^	930		
Ammoniated barley^c^		943	
Palm oil	20	20	
Soy husks	20	20	
Urea	13		
Calcium carbonate	7	7	
Sodium chloride	5	5	
Vitamin mix^d^	5	5	
Composition (g kg^−1^ DM)			
OM	967	954	903
Starch	481	476	26.4
N	24.4	24.3	8.7
NDF	241	252	509
ADF	54.3	59.1	326

^a^ AMM, diet containing ammoniated barley; CTL, control diet.

^b^ Untreated barley, chemical composition (in g kg^−1^ DM): 942 OM, 20.9 N, 199 NDF and 45.9 ADF.

^c^ Ammoniated barley based on the Maxammon process (Harbro Ltd). Chemical composition (in g kg^−1^ DM): 967 OM, 25.4 N, 69.8 NDF and 25.4 ADF.

^d^ Mix containing vitamins (in IU kg^−1^): 85.000 A, 20.000 D_3_ and 68 E.

### Rumen microbial fermentation

On days 15, 16 and 17 (phase 2), rumen digesta samples were collected three times per day at 0, 2 and 4 h after feeding to assess the rumen microbial fermentation profile. Rumen contents (approximately 100 g) were sampled through the rumen cannula and filtered through a double layer of muslin. Rumen pH was immediately recorded, and three sub‐samples were taken. The first sample (1.6 mL) was diluted with 0.4 mL of an acid solution (0.5 mol L^–1^ HCl, 200 g L^−1^ metaphosphoric acid containing 0.8 g L^−1^ crotonic acid as internal standard) for volatile fatty acid (VFA) determination. The second sample (0.8 mL) was diluted with 0.2 mL of trichloroacetate solution (25 g L^−1^) for ammonia analysis and the last sample (0.8 mL) was used to measure lactate concentration. All samples were frozen at −20 °C until further analysis.

### *In sacco* degradability and feed microbial colonization

Two sheep (one from each treatment) were removed from the study at day 21 because of issues unrelated to the treatments (i.e. rumen fluid leaking through the cannula); therefore, five experimental sheep were used thereafter for *in sacco* degradability, digestibility and microbial protein synthesis measurements. An *in sacco* trial (phase 3) was conducted during days 22–25 to analyze rumen degradation kinetics for untreated and AMM incubated in the rumen of sheep fed the CTL and AMM diet, respectively. The *in sacco* nylon bag technique was used as reported previously ^15^: cereals were mill‐ground to pass a 2‐mm screen, and aliquots of each feed (2 g DM) were placed in nylon bags (7 × 10 cm with a 46‐μm pore; Sefar Maissa, SA, Barcelona, Spain), which were incubated for 0, 4, 8, 16 and 24 h in the rumen of each cannulated sheep. Two bags per sheep and incubation time were used. Bags for different time points were placed into the rumen together and removed separately, making sure that no more than 10 bags were incubated at a time. After incubation, bags were rinsed with cold water in a washing machine without detergent for 15 min to minimize the bacterial population attached to the bag feed residues. This rinsing procedure was also used to determine the feed soluble fraction (0 h incubation). Incubated bags were dried at 60 °C for 48 h to measure DM disappearance and feed residues were further used for organic matter (OM) and N analyses.

It was hypothesized that feeding the AMM diet, compared to the CTL diet, could modulate the rumen microbial environment with positive indirect effects on the microbial colonization and degradation of two agro‐industrial by‐products. Orange pulp and tomato‐waste silage were chosen as being among the most commonly used by‐products in Spain but having different chemical composition (in g kg^−1^ DM): 936 and 897 for OM, 8.5 and 19 for N and 17 and 52 for neutral detergent fiber (NDF), respectively. Therefore, during the days 26, 27 and 28 (phase 4), these by‐products were incubated *in sacco* in the rumen of AMM and CTL sheep for 0, 0.5, 1, 2, 4, 8 and 24 h, as described before. Earlier and more frequent sampling (0.5 and 1 h) was performed to improve the resolution during the early microbial colonization.^16^ Incubation bags were then rinsed twice with saline solution (NaCl 9 g kg^−1^) to prevent microbial cell lysis^16^ and snap frozen in liquid N. Samples were freeze dried and DM disappearance and microbial colonization of the feed was assessed based on the DNA quantification.

### Digestibility and microbial protein synthesis

Sheep fed CTL and AMM diets were housed in individual metabolism crates from day 29 to 33 (phase 5). Feed intake was recorded daily and aliquots representing 200 mg L^−1^ total faces and feed refusals were collected from each animal, pooled and analyzed to determine the total tract apparent digestibility. Total urine excretion was collected in containers with 50 mL of sulphuric acid (100 mL L^−1^) to prevent N evaporation. Urine aliquots (100 mL L^−1^) were collected daily and frozen for quantification of creatinine and purine derivatives (PD) as indicators of the rumen microbial protein synthesis.

### Sample analysis

To describe the rumen fermentation, VFA concentration was measured by a gas chromatography system coupled with a flame ionization detector (Auto‐system Perkin‐Elmer Corp., Shelton, CT, USA) as previously described.^4^ Ammonia and lactate concentrations were determined using colorimetric methods.^17^, ^18^ To determine feed degradability and total tract digestibility, samples of feeds, orts, incubation bags and fecal samples were pooled per animal, mill‐ground (1‐mm screen) and analyzed for DM (method 934.01), OM (method 942.05) according to the AOAC.^19^ The N values (method 990.03) were determined by the Dumas method (Leco TruSpec CN, St Joseph, MI, USA). NDF and acid detergent fiber (ADF) were measured as described by Van Soest *et al*.,^20^ using an Ankom 220® fiber analyzer (Ankom Technology Corp., Macedon, NY, USA), with α‐amylase, and NDF and ADF were expressed including residual ash. Starch concentration in the feed was measured using the Total Starch Assay Kit (AA/AMG; Megazyme, Bray, Ireland).

To assess the feed rumen degradation and microbial colonization of the agro‐industrial by‐products, bag feed residues were freeze‐dried and DM disappearance was calculated. Samples were bead‐beaten for 1 min (Mini‐BeadBeater; Biospect Products, Bartlesville, OK, USA) and DNA was extracted using a commercial kit (QIAmp DNA Stool Kit, Qiagen Ltd, Barcelona, Spain) as described previously.^21^ Total DNA concentration was measured using the Nanodrop ND‐100 spectrophotometer (Thermo Fisher Scientific, Waltham, MA, USA) and used as an indicator of the feed colonization by the rumen microbes. Total bacterial concentration was also determined in the feed residues by a quantitative polymerase chain reaction (PCR)^22^ using the 16S rRNA primer sets: forward GTGSTGCAYGGYTGTCGTCA and reverse ACGTCRTCCMCACCTTCCTC. Cycling conditions were 95 °C for 5 min; 40 cycles of 95 °C for 15 s, 60 °C for 30 s and 72 °C for 55 s; and 72 °C for 1 min using a Real‐Time PCR Detection System (Bio‐Rad Laboratories Inc., Hercules, CA, USA). Bacterial DNA standards were generated from a mix of genomic DNA extracted from solid‐associated bacteria as described previously^23^ and serial dilutions (from 10^1^ to 10^6^) were conducted to stablish the calibration curve. Bacterial absolute concentration was expressed as DNA copies mg^−1^ DM in the bag residue.

Urinary excretion of PD (i.e. xanthine, hypoxanthine, uric acid and allantoin) was determined in accordance with the procedure described by Balcells *et al*.^24^ In brief, a high‐performance liquid chromatography equipped with a multi‐solvent delivery system (model 5110; Hitachi Chromaster, Tokyo, Japan) and an auto‐sampler (model 5210) was used. A double 4.0 × 250 mm C18 ODS‐2 analytical column (Waters Spherisorb®; Waters Corp., Dublin, Ireland) was used for PD separation, and detection was made with a diode array detector (model 5430; Hitachi Chromaster) in the wavelength range 190–250 nm, set at 205 nm for quantification. Calibrations were made with serial dilutions of each pure PD and allopurinol was used as internal standard.

### Statistical analysis

*In sacco* degradation profiles were calculated by the nonlinear model described by Orskov and McDonald.^25^ The effective degradability (ED) in the rumen was calculated, using the nonlinear regression procedure as:

$$\mathrm{ED}=a+\mathrm{bc}/\left(c+k\right)$$

where *a* is the water‐soluble fraction, *b* is the potentially degradable insoluble fraction, *c* is the fractional degradation rate of *b* per hour and *k* is the passage rate of the digesta out of the rumen, which was assumed to be 0.02 h^−1^ for sheep fed at maintenance level.^26^ Total DNA and bacterial concentrations were log10‐transformed to obtain normal distributions. The effect of the treatment (CTL *versus* AMM) on *in sacco* degradability, digestibility and PD data was analyzed by one‐way analysis of variance. For rumen fermentation data, averages of the three consecutive days were calculated for comparable time points within each sheep and analyzed using a repeated measures mixed‐effects model (residual maximum likelihood) via SPSS, version 21.0 (IBM Corp., Armonk, NY, USA) as:

$$Y_{\text{ijklm}}=\mu +A_{i}+T_{j+}\;{\left(A\times T\right)}_{\mathrm{ij}}+S_{k}+e_{\text{ijkl}}$$

where *Y*_*ijkm*_ is the dependent, continuous variable, *μ* is the overall population of the mean, *A*_*i*_ is the fixed effect of the cereal ammoniation treatment (*i* = CTL *versus* AMM), *T*_*j*_ is the fixed effect of the sampling time (*j* = 0 *versus* 2 *versus* 4 h after feeding), *(A × T)*_*ij*_ is the interaction term, *S*_*k*_ is the random effect of the sheep (*k* = 1 to 5) and *e*_*ijkl*_ is the residual error. This repeated measures analysis was also used for feed intake and rumen pH data but considering different time scales (weeks 1 to 5 for feed intake and 0, 2, 4, 6 and 24 h for rumen pH). When significant effects were detected, means were compared by Fisher's protected least significant difference test. *P* < 0.05 was considered statistically different.

## RESULTS

### Feed intake and rumen fermentation

**Figure 2:**
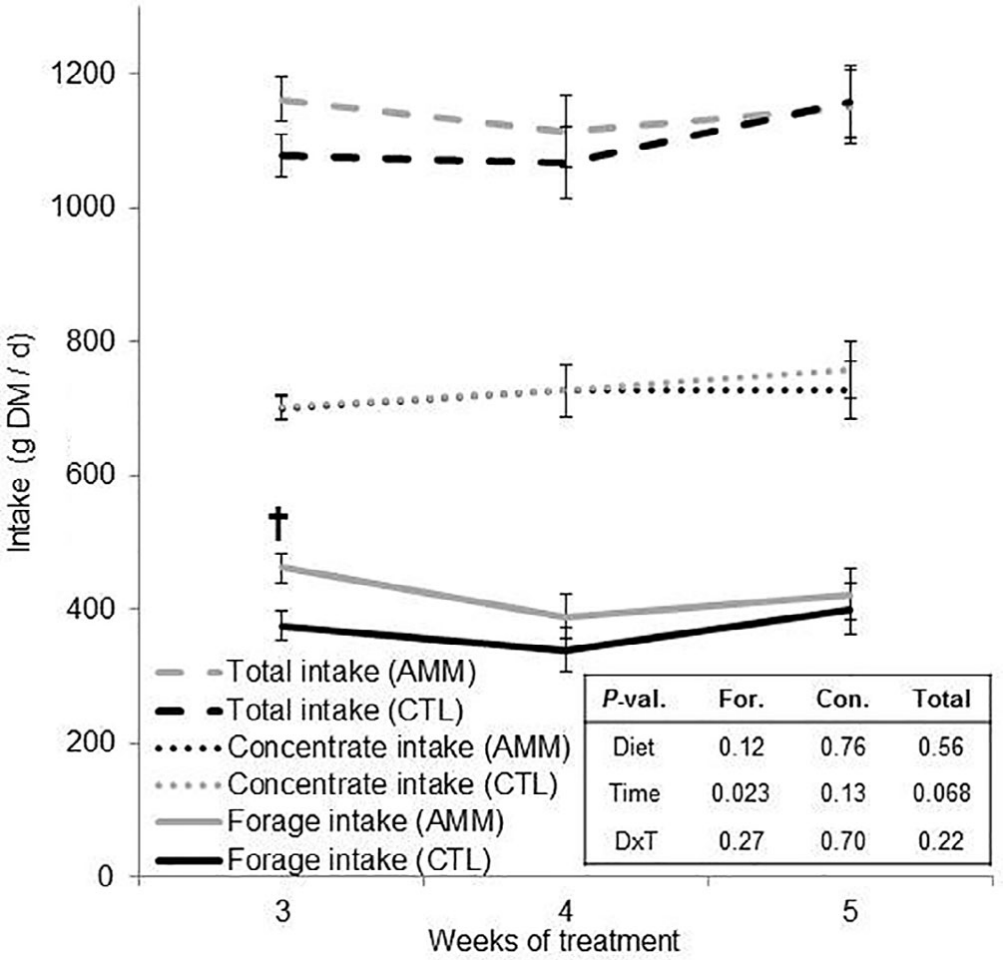
Effect of barley ammoniation on average feed intake. CTL, control diet; AMM, diet containing ammoniated barley. †*P <* 0.1. Bars indicate the SEM.

Sheep remained in good health and did not show signs of illness throughout the experiment. Sheep fed the AMM diet, compared to the CTL diet, tended to have higher forage intake during week 3 (*P <* 0.1) (Fig. 2) although the average forage intake over the whole experiment did not show significant differences (453 *versus* 406 g day^−1^). No differences were noted in concentrate intake and total DM intake between treatments.

**Figure 3:**
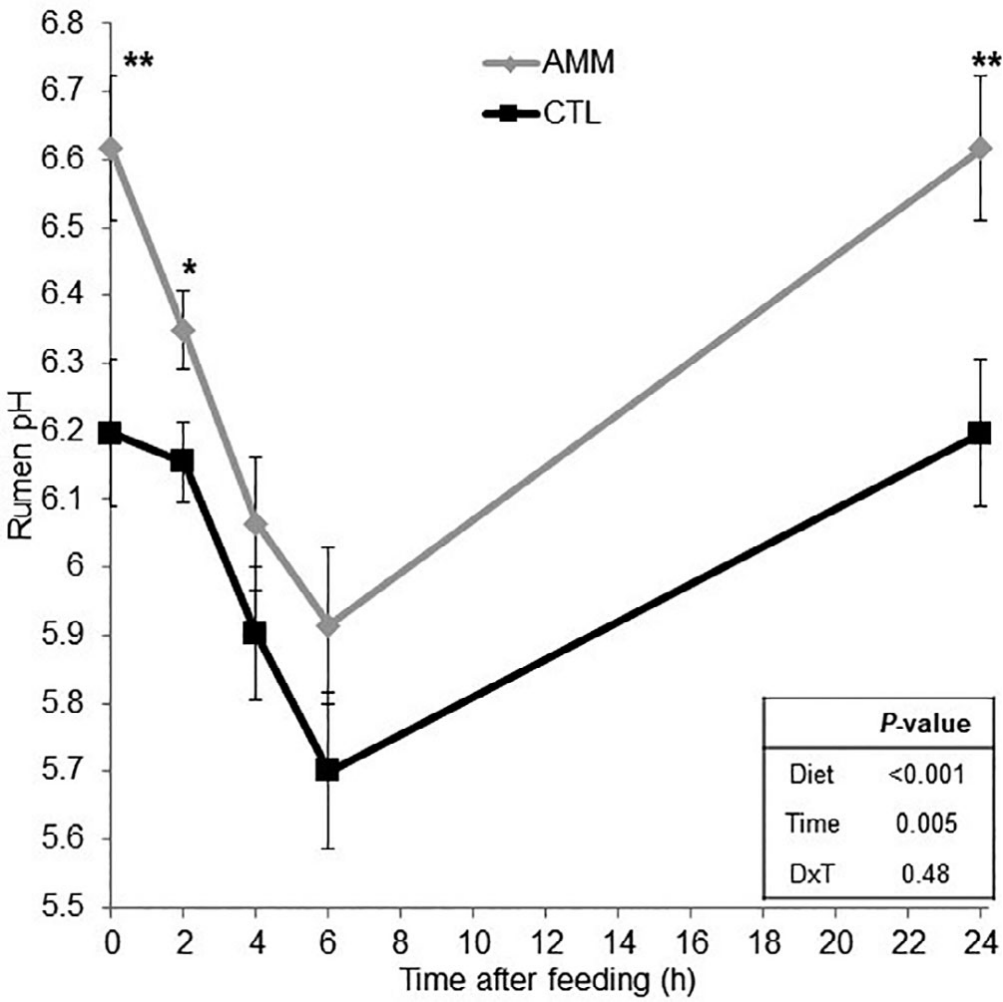
Effect of barley ammoniation on rumen pH. CTL, control diet; AMM, diet containing ammoniated barley. *P < 0.05; **P < 0.01. Bars indicate the SEM.

Rumen pH values showed a sharp decline after feeding (*P <* 0.001) (Fig. 3) and AMM sheep had significantly higher rumen pH than CTL sheep (6.24 *versus* 5.99, *P* = 0.009). No significant interactions were observed between the diet and the sampling time in relation to rumen fermentation variables; therefore, only the fixed effects were reported (Table 2). Rumen ammonia‐N concentration peaked at 2 h after feeding (*P =* 0.001), and this peak tended to be more evident for CTL than for AMM diets (296 *versus* 186 mg N L^−1^), as indicated by the interaction between diet and time (*P* = 0.098). Lactate and total VFA concentrations peaked at 4 h after feeding (*P <* 0.01) with no differences between CTL and AMM diets. Sampling time also affected the VFA profile and the AMM diet tended to promote a higher molar proportions of acetate (*P =* 0.070) than the CTL diet.

**Table 2:** Effect of barley ammoniation and post‐feeding time on rumen fermentation in sheep

	Diet		Time after feeding				*P*‐value		
Treatment^†^	CTL	AMM	0 h	2 h	4 h	SEM	Diet	Time	D × Time
Ammonia‐N (mg L^−1^)	167	127	95.5 a	241 b	103 a	66.19	0.33	< 0.001	0.098
Lactate (μg mL^−1^)	29.9	29.8	28.6 a	25.4 a	35.6 b	3.797	0.98	< 0.001	0.57
Total VFA (mmol L^−1^)	88.9	90.9	85.2 a	85.3 a	99.1 b	7.470	0.62	0.002	0.76
Molar proportion									
Acetate (mmol mol^−1^)	631	664	667 c	630 a	645 b	10.79	0.070	< 0.001	0.56
Propionate (mmol mol^−1^)	197	188	172 a	206 b	199 b	12.30	0.61	< 0.001	0.57
Butyrate (mmol mol^−1^)	129	114	117 a	127 b	120 a	5.476	0.25	0.005	0.94
Isobutyrate (mmol mol^−1^)	8.89	8.50	10.6 b	8.33 a	7.17 a	11.67	0.63	< 0.001	0.72
Valerate (mmol mol^−1^)	20.5	18.5	19.5	19.6	19.4	1.430	0.29	0.97	0.49
Isovalerate (mmol mol^−1^)	14.1	6.94	13.8 b	9.08 a	8.75 a	1.171	0.15	< 0.001	0.53
Acetate:propionate	3.31	3.67	4.01 b	3.11 a	3.35 a	0.270	0.37	< 0.001	0.32

Means with different lowercase letters within a row differ for the time effect (*P* < 0.05).

^†^ AMM, diet containing ammoniated barley; CTL, control diet.

### *In sacco* degradability

No differences were noted between untreated and AMM in terms of OM degradability kinetics (Table 3). However, this ammoniation treatment increased the total N content (25.4 *versus* 20.9 mg kg^−1^ DM) (Table 1), which led to higher values for fractions *a* (*P* < 0.001), *b* (*P* = 0.004) and ED (*P* < 0.001) when expressed as mg N kg^−1^ DM (Table 3). AMM also showed a higher fraction *a* (*P* < 0.001) and value *c* (*P* = 0.001) when expressed as a proportion of the total N, whereas untreated barely had higher fraction *b* proportion (*P* = 0.010), with no differences in terms of ED.

**Table 3:** Effect of barley ammoniation on rumen OM and N *in sacco* degradability

Treatment	Untreated barley	Ammoniated barely	SEM	*P*‐value
OM degradability				
*a* (g kg^−1^ OM)	237	236	1.147	0.370
*b* (g kg^−1^ OM)	659	662	15.41	0.894
*c* (h^−1^)	0.076	0.088	0.007	0.254
ED (g kg^−1^ OM)	872	870	16.40	0.933
*a* (g kg^−1^ DM)	229	222	2.411	0.050
*b* (g kg^−1^ DM)	638	624	14.85	0.536
ED (g kg^−1^ DM)	844	820	15.77	0.322
N degradability				
*a* (g kg^−1^ N)	349	399	1.605	< 0.001
*b* (g kg^−1^ N)	565	517	9.786	0.010
*c* (h^−1^)	0.060	0.082	0.003	0.001
ED (g kg^−1^ N)	897	895	10.34	0.893
*a* (g kg^−1^ DM)	7.29	10.1	0.034	< 0.001
*b* (g kg^−1^ DM)	11.8	13.1	0.218	0.004
ED (g kg^−1^ DM)	18.7	22.7	0.229	< 0.001

**Figure 4:**
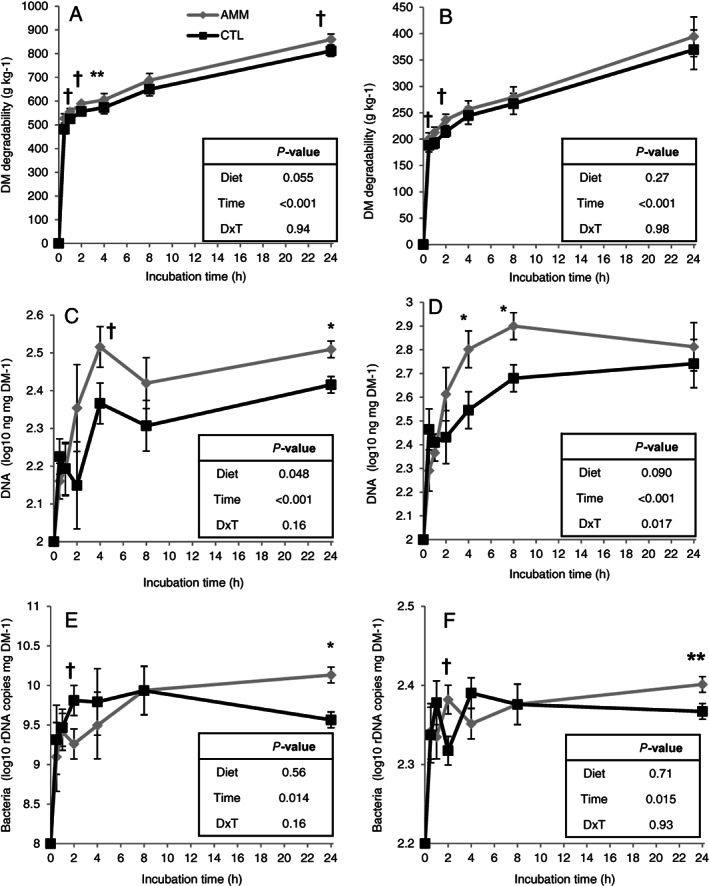
Effect of barely ammoniation of the basal diet on *in sacco* DM degradability and microbial colonization of orange pulp (A, C and E) and tomato‐waste silage (B, D and F). CTL, control diet; AMM, diet containing ammoniated barley. †*P <* 0.1, *P < 0.05; **P < 0.01. Bars indicate the SEM.

Feeding the AMM diet generated a rumen microbial environment which, to some extent, modified the *in sacco* degradation and microbial colonization pattern of two agro‐industrial by‐products (Fig. 4). Orange pulp and tomato‐waste silage showed a progressive increase in the DM degradation over time, which was accompanied by similar increments in the total DNA and microbial DNA concentrations (*P* < 0.05). Orange pulp incubated in the rumen of sheep on the AMM diet, compared to those fed the CTL diet, showed a tendency for higher *in sacco* DM degradability values (*P =* 0.055) and a greater total DNA concentration in the residual feed throughout the incubation process (*P =* 0.048). Tomato‐waste silage incubated in the rumen of AMM sheep also had higher total DNA concentration in the feed residue but only during the 6–8‐h interval (interaction, *P =* 0.017), whereas it only tended to have higher DM degradation values during the 2–4‐h interval. Quantitative PCR data showed no differences between AMM and CTL sheep in the overall feed bacterial colonization, although both by‐products reached higher bacterial DNA concentration after 24 h of incubation in the rumen of AMM compared to in CTL sheep (*P <* 0.05).

### Digestibility and microbial protein synthesis

No differences between diets were observed for the total tract feed digestibility (Table 4), although AMM sheep tended to have higher N digestibility than CTL sheep (*P =* 0.096). Urinary excretion of creatinine was similar across treatments when expressed either per day or by metabolic body weight. By contrast, the urinary excretion of total PD was greater for AMM than CTL sheep when expressed per day (*P* = 0.039) or by metabolic body weight (*P =* 0.031) but not as a PD:creatinine ratio (*P =* 0.16).

**Table 4:** Effect of barley ammoniation on total tract apparent digestibility and urinary excretion of purine derivatives (PD)

Treatment^a^	CTL	AMM	SEM	*P*‐value
Apparent digestibility (g kg^−1^)				
DM	625	628	21.8	0.92
OM	689	669	29.5	0.64
NDF	479	434	26.0	0.26
N	475	579	19.6	0.096
Urinary excretion				
Creatinine (mmol day^−1^)	8.30	8.87	1.03	0.70
Creatinine (mmol kg^−1^ BW^0.75^)	411	460	56.9	0.57
PD (mmol day^−1^)	6.69	8.97	0.65	0.039
PD (mmol kg^−1^ BW^0.75^)	332	461	35.1	0.031
PD: creatinine ratio	0.82	1.08	0.12	0.16

^a^ AMM, diet containing ammoniated barely; CTL, control diet.

## DISCUSSION

### Feed intake and rumen fermentation

The identical and progressive increase over time of concentrate intake in CTL (containing urea) and AMM sheep during the first 2 weeks indicated that they may need an adaptation period to get used to the bitter taste associated with urea.^27^ Once adapted, the tendency for greater forage intake (+14% on average) observed in AMM sheep from weeks 3 to 5 may indicate a more favorable rumen environment for fiber degradation.^23^ However, these feed intake data should be carefully interpreted given the inherent variability associated with the frequent manipulation of the experimental sheep. AMM sheep had higher rumen pH values before the morning feeding (6.62 *versus* 6.20) and remained higher through the day, with values always above 6.0. This higher rumen pH could be partially explained by the higher pH reported for ammoniated than for untreated grains,^28^ although other indirect factors such as changes in the rumen microbiota could be also implicated in this process. By contrast, CTL sheep showed pH values below 6.0 at 4 and 6 h after feeding, indicating a higher risk of experiencing rumen acidosis.^29^ Robinson and Kennelly^11^ reported that the post‐feeding decline in ruminal pH was negatively correlated to the ammoniation levels of barley, with this response being attributed to the slower rate of starch degradation in the rumen. It has been reported that incubations with rumen fluid at a constant pH of 6.5 promoted higher degradation of starch than those with lower pH,^30^ suggesting that high pH positively affects amylolytic bacteria, as noted in the present study. Similarly, several studies have suggested that high pre‐prandial rumen pH positively influences the attachment of fibrolytic bacteria (e.g. *Fibrobacter succinogenes* and *Ruminoccocus albus*) to the feed and feed degradation.^31^, ^32^, ^33^ As a result, it has been suggested that maintaining the pH above 6.0 is important for an efficient microbial attachment and fiber digestion in the rumen.^31^ Although our experiment did not investigate the rumen microbiota, the higher rumen acetate concentrations (+5.1%) indicated a more active cellulose and hemicellulose degradation in the rumen.^34^ A recent study, in which the same cereal ammoniation process as that tested here was used,^12^ noted that dairy cows fed a diet supplemented with 15.5% of ammoniated grains (triticale and oats), compared to non‐ammoniated cereals, had higher rumen pH (5.98 *versus* 5.66), total VFA concentration (+15%) and acetate molar proportion (+6.4%), resulting in an increase in energy corrected milk yield. This later work suggested that the higher rumen pH in animals fed ammoniated cereals was mostly attributed to its alkalinizing effects in the rumen and the slower starch degradation rate. This hypothesis was supported by the lower ruminal levels of *Streptococcus bovis*, a lactate producer which has been associated with ruminal acidosis,^35^ and higher levels of lactate utilizers such as *Selenomonas ruminantium* and *Megasphaera elsdenii* as a mechanism to prevent rumen acidosis.^36^

Feed‐grade urea is an inexpensive source of N that supplies soluble N for ruminal microbes; however, as a result of the rapid conversion of urea to ammonia in the rumen, and the potential associated problems, there is a growing interest in slowing down this process.^8^ In the present study, the concentrations of rumen ammonia‐N were always above the critical values (50 mg L^−1^) to ensure optimal microbial activity in the rumen.^37^ In this sense, despite both diets being iso‐nitrogenous and formulated to have equivalent amounts of non‐protein N, AMM treated sheep maintained a narrow range in rumen ammonia‐N concentration (from 80 to 186 mg L^−1^), whereas CTL sheep had a larger post‐prandial variation (from 76 to 296 mg L^−1^). As a result, CTL sheep had 60% higher rumen ammonia‐N concentration at 2 h after feeding than AMM sheep. In this sense, we have previously shown that preventing an excessive post‐prandial ammonia peak is associated with improved N incorporation by the rumen microbes and higher N use efficiency in dairy cows,^38^ as noted in the present study.

### Rumen degradability and total tract digestibility

Rode *et al*.^10^ reported coefficients of starch digestibility in steers of 77%, 69% and 69% in ammonia‐treated, urea‐treated and whole moist barley, respectively, suggesting that ammoniation exerts a positive effect on feed degradation. In the present study, the positive effects of cereal ammoniation on rumen pH and forage intake were not translated into superior rumen OM degradability or total tract apparent digestibility for DM, OM and NDF. The low feeding level used in the present study (around 1.2 times maintenance energy requirements) was chosen to minimize the inter‐animal variation, although it could also have minimized the dietary impact on total tract digestibility. It might be hypothesized that, under higher feed intake levels (as seen in dairy cows or beef cattle), the positive impact of high rumen pH on feed digestibility could be greater because the rumen retention time is lower and can limit feed utilization by the microbes.^31^

By contrast, the cereal ammoniation treatment had some positive effects on the rumen N metabolism. Barley ammoniation led to a substantial increase in N content (+21.5%) compared to untreated barley. This increase in total N content was translated into an equivalent increase in the effective degradable N content (+21% in DM) as a result of its higher soluble N (38%) and potentially degradable insoluble N content (+11%) than that observed in untreated barley. If urea was added to barley to obtain a similar increment in the total N content, a greater increase in the soluble N content (+48%) would be expected because of its high solubility, resulting in a greater ammonia postprandial peak as noted in this study. These findings suggest that part of the non‐protein N added into the AMM (approximately 29%) is not immediately available for the rumen microbes, showing comparable N degradation pattern (although with a higher fractional degradation rate) than that observed for the barley protein N. It has been hypothesized that ammonia captured within a cellular matrix of ammoniated cereals might be released more slowly than free ammonia added to the diet in form of urea in the CTL diet.^9^ These findings are in line with the observed tendency for a higher N total tract apparent digestibility (+21%) in the AMM compared to the CTL diet, possibly as a result of a more efficient N uptake in the rumen^39^ and/or a higher N uptake in the intestine, as suggested previously in dairy cows^11^ and feedlot cattle.^40^ However, some studies have reported negative effects of AMM on feed digestibility in beef cattle,^41^ suggesting that this treatment, *per se*, may not be as effective as mechanical processing with respect to improving daily gain or feed efficiency, and the most promising results are observed when ammonia treatment is combined with mechanical^42^ or enzymatic processes, as used in the AMM diet.^12^, ^13^

In the present study, we tested two by‐products with high potential use in the Mediterranean basin: orange pulp from juice making^43^ and silage made with tomato‐waste pulp and straw.^4^ The results obtained indicated that the AMM diet could generate a rumen microbial environment that favored, to some extent, the utilization of these by‐products by the rumen microbes. Despite a great variability being noted as a result of the high rumen sampling manipulation during the early colonization (0.5–4‐h interval), the tendency to higher *in sacco* DM degradation observed in AMM compared to in CTL sheep for the orange pulp (+6.3%) is in line with its higher feed microbial colonization detected from 4 h after feeding in terms of total DNA (+6.8%) and in terms of bacterial DNA at 24 h after feeding. Most of the non‐microbial DNA is rapidly degraded in the rumen, As a result of previous studies using ^15^N as a microbial marker to describe the feed colonization process, it has been revealed that higher levels of total DNA and/or bacterial DNA are indicators of the feed microbial colonization, which is linked with higher rates of feed utilization (particularly for fibrous feeds) by the microbes.^16^, ^44^ Because the different rumen microbes (i.e. bacteria, methanogens, protozoa and fungi) have different feed colonization patterns,^16^ the total DNA concentration data gave an insight to the overall feed microbial colonization, whereas the quantitative PCR data provided a more specific view of the bacteria as the preponderant microbial group in the rumen. Thus, our observations suggested that feeding ammoniated cereals can have positive indirect effects on the utilization of some alternative fibrous feeds by accelerating their microbial colonization, possibly as a result of higher rumen pH^31^, ^32^ and/or improved synchrony in the availability of energy and N for the rumen microbes.^45^ These improvements in feed microbial colonization were less obvious for the tomato‐waste silage than for the orange pulp, possibly as a result of the higher N (+125%) and NDF (+196%) contents, which could result in a less limiting N availability for the fibrolytic bacterial growth.^38^

### Microbial protein synthesis

Urinary creatinine excretion values were in the range published for sheep fed at maintenance level,^46^ indicating that a total collection of urine was achieved, and that sheep did not experience changes in body weight and composition. The higher PD excretion observed in AMM compared to in CTL sheep when expressed per day (+34%), per metabolic weight (+39%) or as change in the ratio PD:creatinine (+31%) suggested that AMM treatment enhanced the flow of microbial protein to the duodenum. This greater microbial protein synthesis may be related with the improved rumen microbial growth (as noted by the higher total DNA and bacterial DNA associated with the feed) and could indirectly explain the higher total tract N digestibility observed for AMM diet as a result of the higher availability of slow release N for the rumen microbes. Rumen protozoa are bacterial predators and have a negative impact on the microbial proteins synthesis and N use efficiency by the animal.^47^ Therefore, the higher microbial protein synthesis observed in our AMM compared to in CTL sheep is in line the lower rumen protozoal and increased bacterial concentration reported in dairy cows supplemented with ammoniated cereals to partially replace soybean meal as N source.^12^ This observation could explain the greater energy corrected milk yield (+8.6%) and milk protein content (+4.6) observed in cows supplemented with ammoniated cereals compared to in those supplemented with untreated cereals and soybean meal.^12^ Similar improvements in milk yield (+12%) and feed efficiency (+11%) were reported in dairy cows^11^ and feedlot beef cattle fed ammoniated cereals,^13^ indicating that this nutritional strategy could help to improve farm sustainability by decreasing the use of imported protein sources and increasing livestock productivity.

## CONCLUSIONS

Feeding diets including AMM to sheep, instead of untreated barely supplemented with urea, promoted a healthy rumen function with pH values within a physiological range and more efficient use of N by the rumen microbes. This nutritional strategy increased the microbial protein reaching the duodenum and the N digestibility, which together could favor animal performance under intensive production systems. This cereal ammoniation process also accelerated the utilization of agro‐industrial by‐products by rumen microbes, which, along with the improved used of non‐protein N, could help to decrease the environmental impact of ruminant production.

## CONFLICT OF INTERESTS

The authors declare that they have no conflicts of interest.
